# Experience-based co-design (EBCD) with young people who offend: Innovating methodology to reach marginalised groups

**DOI:** 10.1371/journal.pone.0270782

**Published:** 2022-07-12

**Authors:** Melissa Girling, Ann Le Couteur, Tracy Finch

**Affiliations:** 1 Population Health Sciences Institute, Newcastle University, Newcastle, United Kingdom; 2 Department of Nursing, Midwifery & Health, Faculty of Health & Life Sciences, Northumbria University, Newcastle, United Kingdom; Universitat Luzern, SWITZERLAND

## Abstract

The mental health needs of young people who offend have become more widely recognised and attempting to meet these needs is now a global priority for governments and health agencies. Young people who offend experience a range of complex difficulties and have significantly worse health and social outcomes than their mainstream counterparts. These problems usually persist and often increase in severity through adolescence and into later life. There is growing acceptance of the potential value of co-designing services that recognise and address problems to improve the outcomes of young people with mental health problems yet to date, this methodological approach remains relatively unexplored in forensic service provision. Experience-based co-design (EBCD) is an approach to healthcare improvement that enables staff and service users to jointly co-design services. Central to the approach is the idea that understanding the experiences of service users and the ‘touchpoints’ (e.g., critical points or moments) in their journey through a service are integral to service improvement. The aim of this study was to explore whether EBCD could be applied to facilitate recognition of, and service developments for, young people presenting in community forensic settings. Qualitative methods used in this study included: observational fieldwork in four police custody suites (n = 30 hours), in-depth interviews with staff in community forensic services (n = 13) and researcher staff (n = 7). In this paper, the challenges of applying EBCD in community forensic settings with this population were: working with and across agencies; gaining access to participants; understanding knowledge and power dimensions amongst participants and understanding the context. This paper argues that innovative approaches to discovering the touchpoints for young people who offend – a key component of the EBCD approach - through combining analyses of secondary data and direct observations in community forensic settings can facilitate engagement with these specialist services and so provide access to relevant information about a group (i.e., young people who offend) who may be unable to participate directly in the EBCD process.

## Introduction

In England and Wales it is estimated that 1 in 10 young people aged 5-16 has a mental disorder [1] and certain groups of young people, e.g. those who offend (10-18 years), experience significantly more mental health problems and are over represented in the justice system [2]. Young people who offend are approximately three times more likely than non-offenders to experience mental health problems [3] and 95% of young people aged 16 to 20 in England and Wales Young Offender Institutions (YOIs) experience one or more mental disorders [4]. In addition, 25-50% of young offenders have some form of learning disability [5, 6] and/or speech, language or communication need [7, 8].

The health and well-being of young people who offend has increasingly been identified as a policy priority in England and Wales [8–11]. The recent ‘Future in Mind’ Government strategy aimed at promoting and improving the mental health of all children and young people, recognises the need for an integrated system to meet the needs of particularly vulnerable groups such as those involved in the youth justice system. To achieve this, the strategy describes a need for effective partnerships between for example, mental health services and existing services in other agencies such as the youth justice system together with a recognition of the importance of including young people as ‘experts in their care’ [12]. In part response to this strategy, in 2015 the UK Government announced a comprehensive review of the youth justice system. The review was tasked with (i) determining whether or not the current system is ‘fit for purpose’ and (ii) assessing ways in which a more effective ‘joined-up’ system between children and young people’s services could potentially operate [13]. Recommendations from the review include the devolution of Youth Offending Teams (YOTs) to local authorities and re-organising mental health support for those at risk in line with the increased funding for young people’s mental health services more broadly [14]. However, these recommendations did not sufficiently acknowledge the complex needs of young people who offend, nor identify ways to listen to and act upon the views and experiences of these vulnerable young people. In their joint response to the review, voluntary sector organisations argued that improving outcomes for young people who offend requires a more nuanced understanding of the types of interventions that are most likely to meet their needs [15].

Despite a growing acceptance of the potential value of co-designing services to recognise and address problems [12], few studies have investigated how best to ascertain the experiences of young people who offend and what impact this information might have on service provision or on the outcomes for this group [16]. This paper reports findings from a multi-component study which had two purposes namely to apply the experience based co-design (EBCD) methodology to generate understanding of the experiences/needs of participants (young people and employed staff) and to investigate the use of the EBCD methodology in a previously unexplored context i.e., community forensic services. This paper focuses primarily on the latter i.e., the use of EBCD methodological process in community forensic settings for young people who offend and understandings gained from this. The co-design generated findings relating to young people who offend and professionals working in this context that will be reported separately [17]. Data relating to findings about the participants are therefore used only for the purpose of illustrating methodological issues that relate to some of the legal, ethical, and practical challenges of, as well as possible solutions to, applying the approach to engage with a population that is infrequently heard.

## Methods

### Experience-Based Co-Design (EBCD)

Experience-based co-design (EBCD) was first developed by Bate and Robert in 2006 as an innovative approach for use in healthcare improvement research. The aim of the approach is to guide service improvement through staff and service users working together collaboratively to co-design better services [18]. Central to this approach is the notion that experiences held by service users are unique and integral to the process [18].

The EBCD approach has two phases: an exploratory phase and a co-design phase. The first exploratory or discovery phase is rooted in anthropology and ethnography and differs from traditional qualitative approaches to healthcare improvement research in that Bate and Robert describe it is a ‘joint venture’ between staff and patients. It places emphasis on the actual experiences of staff and service users rather than their views, attitudes or needs [19]. Through exploring the experiences of staff and service users, the EBCD approach aims to create services that are ‘cognitively and emotionally’ appealing to those who receive them [19]. The second co-design phase is focussed on service users actively and directly participating in specific parts of, or the whole design process itself. In its traditional form the EBCD approach involves six-defined stages and is typically undertaken over a period of 9-12 months [19].

The six stages involve a process of:

1. Setting up the project and getting staff ‘buy in’ to the project
2. Gathering staff experiences through a combination of semi-structured interviews (n = 12-15), periods of participant observation in services and presenting this information to staff participants
3. Gathering the experiences of service users (n = 12-15) through open-ended narrative interviews which are typically filmed, and feeding back this information to service user participants
4. Bringing participants together in a co-design event to identify emotionally significant points or ‘touchpoints’ where services users come into contact with services and to develop a set of key priorities to address
5. Organising small groups in which staff and service user participants work together to co-design improvements to the priorities jointly identified in stage 5
6. Holding an end of project celebration event to reflect on what has been achieved and identify areas for further consideration [19, 20].

Bate and Robert describe how undertaking periods of direct observation in particular (a feature of stage 1) can provide important understanding about the ways in which staff and service users interact with each other and their environment ‘in real time’ [19]. For example, observations can highlight discrepancies between the accounts of what people say and what they do [21] and can reveal previously unconsidered ‘touchpoints’ [19] and facilitate engagement with staff [20].

### The original proposed EBCD study

The original study designed to generate understanding of the experiences/needs of participants (young people who offend and employed staff) was approved by Newcastle University’s Research Ethics Committee (Ref: 1230/10204/2017). Informed verbal consent was obtained for observational aspects of the study and informed written consent was obtained for participant qualitative interviews. The original study aimed to follow the components of the traditional EBCD approach (see Box 1). However, throughout the development and preliminary phases of the study, significant challenges were experienced accessing and recruiting young people who offend. Several staffing (both managerial and organisational) changes occurred at the community forensic study sites. For example, some senior level staff left their posts due to promotion, secondment, redundancy, or retirement. This led to difficulties maintaining consistent working relationships with, and ‘buy in’ from the services. Participation of staff in some research stages (including agreement to facilitate research access to young people who offend; and engagement in feedback and co-design events) was no longer possible due to unavoidable resource constraints (e.g., redundancy) and other factors such as, Youth Offending Teams (YOTs) timetables and changes in police/custody shift patterns that prevented the agreed and pre-planned recruitment of young people. Thus, despite the prior agreement of the senior management teams and the use of several recruitment strategies over a six-month period no young people were recruited.

**Box 1. The original proposed EBCD study.<Box_Caption**

**The modified EBCD methodological study**

**Fig 1:**
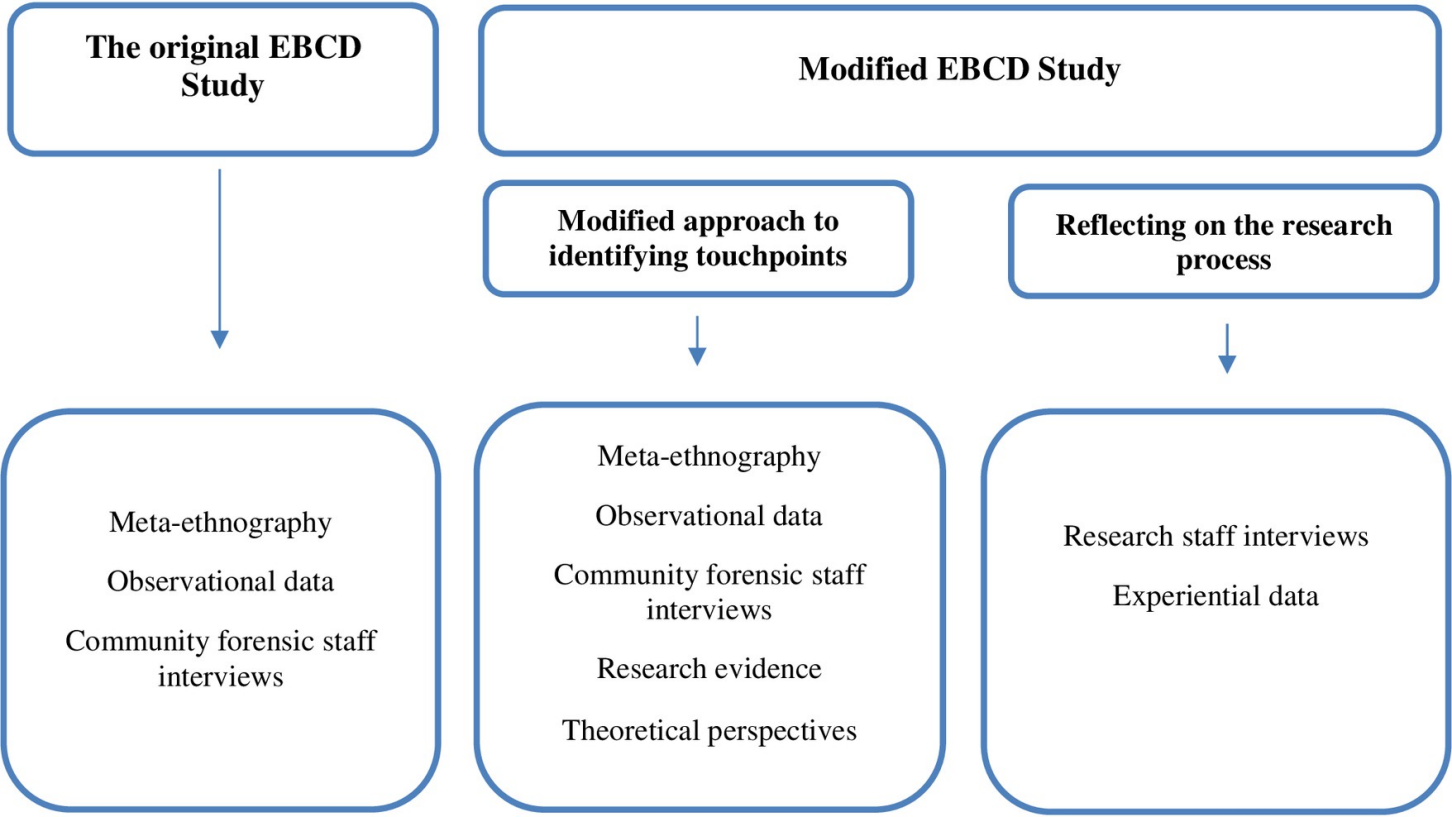
Proposed and modified study methods and data sources.

With the aim of mitigating the recruitment and access challenges experienced in the original EBCD study, a revised research plan (Modified EBCD) was developed to focus on: (1) identifying viable data sources that could be used to discover likely touchpoints and (2), adopting a reflexive approach to the research process itself (see Fig 1) as a further data source.

#### 1. Identifying available data sources that could be used to discover likely touchpoints

A range of steps were undertaken to begin to identify available data sources: project team discussions, further analysis of the EBCD methodological literature, exploring innovative approaches and consultation with experts in applying EBCD type methodology, in particular, the co-founder of EBCD Professor Glenn Robert and international researchers who have successfully completed and published studies using EBCD methodologies. As a result, a number of options were identified and careful in-depth consideration and exploration was given to each. Options included approaching different groups of young people to participate (e.g., young people in residential youth offending institutions or third sector organisations). Due to time constraints in the study and the continued high risk of not being able to recruit young people through these avenues (i.e., involving responsible gatekeepers – usually professionals involved in young people’s care), further options were explored. This included a single case study design or recruiting young people who were ex-service users (e.g., ex-offenders or care leavers) who were likely to be older and therefore able to be approached independently and give their own informed consent. However, these young people were also more likely to be out of the youth justice system and their experiences would therefore be based on retrospective rather than contemporaneous accounts. Further options raised a number of conceptual challenges such as, that including young people in residential youth offending institutions would alter the conceptual focus of the research from a study focussing on the experiences of community based young people who offend to those of young people within the secure estate.

Finally, the option to use an accelerated EBCD approach was considered, whereby pre-existing interview transcripts and/or video clips from other similar studies are re-analysed [22]. However, despite extensive searches (e.g., internet searches including data repositories such as healthtalk.org, and discussions with Professor Robert and international researchers in the field of EBCD) no relevant national or international data sources or similar projects were identified. None of the options described were considered realistically feasible for this study. However, the original proposed EBCD study included a meta-ethnography of qualitative research studies exploring young people’s direct experiences of mental health in youth justice services (see Box 1). Despite the limitation of the retrospective nature of some of the qualitative data, it was recognised that this combined sample included verbatim accounts of young people obtained during each of the qualitative research studies included in the review. This secondary qualitative data set provided direct information about a broad range of young people’s experience of mental health in non-resident community youth justice and the opportunity to access the language of the young people in these contexts. Using this data source, the research team were able to identify an initial set of touchpoints through a secondary investigation of previously collected data. This procedure is in keeping with EBCD methodology [22, 23].

#### Procedure used to identify touchpoints

**Fig 2:**
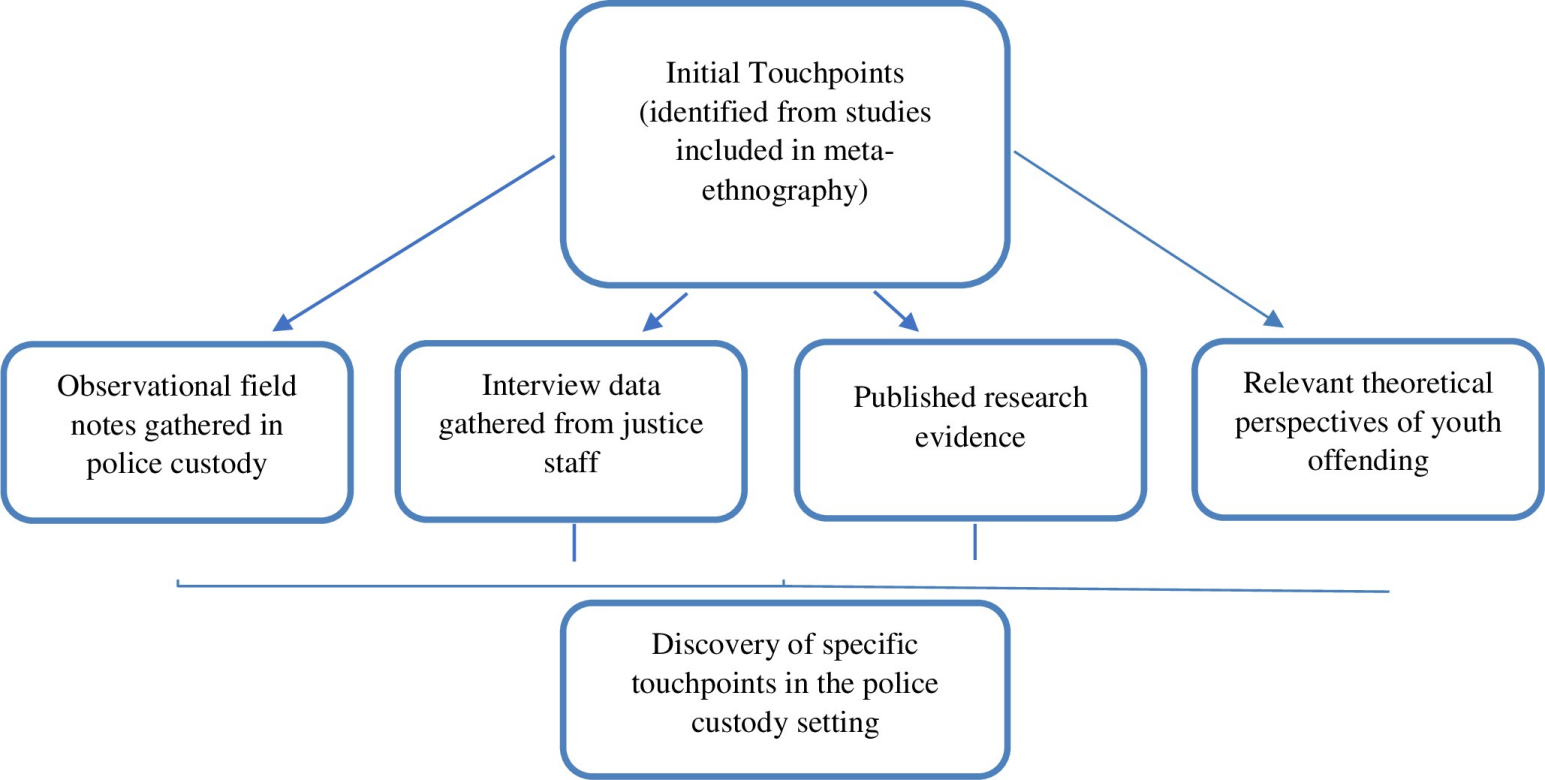
Modified process for developing touchpoints for young people who offend.

The process of identifying touchpoints involved re-reading the pre-determined published data in the original fourteen qualitative studies identified during the meta-ethnography [24] to uncover moments, interactions and events that appeared salient in young people’s experiences (see Fig 2). A number of initial touchpoints were identified which, although not related to young peoples’ specific journey through community forensic services, seemed to be important touchpoints that could apply to their experiences of youth justice as a whole. A procedure was developed (building on consultation with Professor Robert) to use these initial touchpoints as a starting point and then further explore them through a combination of other data sources including: the field notes that were recorded during the observation visits in police custody suites; the qualitative data obtained from interviews with staff in community forensic settings; and relevant theoretical perspectives of youth offending and published research evidence. Direct quotes from young people were extracted from the studies to illustrate the initial touchpoints and to convey young people’s experiences in their own words. Quotes were then placed alongside data from the researcher’s observational field notes that represented similar experiences or instances. A synthesis of all the data sources was then undertaken with the aim of exploring how this might contribute knowledge to what touchpoints in the journeys of young people who offend, through police custody suites might look like and identify possible explanations about why the ‘touchpoints’ might have occurred.

#### 2. Adopting a reflexive approach to the research process

*Researcher interviews*. A further opportunity to gain additional insights into the experiences of service users and providers in community forensic settings was gained by undertaking qualitative interviews with researchers who had previously collected research data in these settings. Potential interviewees (participants) were identified through discussions with the UK expert on EBCD (Professor Glenn Robert) and his professional links to national/international academic research staff who had undertaken modified EBCD studies and through links with a UK regional third sector organisation. These discussions included four academic research staff [ARS] involved in two North American EBCD studies of youth mental health and three service provider staff [SPS] in a third sector organisation involved in UK participatory research work with young people who offend. Participants (researchers) provided written consent to take part in individual, face-to-face, semi-structured interviews. The qualitative interview schedule was developed to address broad issues relating to their experiences of the barriers and facilitators of conducting EBCD/similar participatory studies and ways to better engage with youth in contact with mental health/and or youth justice services. The interviews lasted between 22 and 52 minutes, were audio-recorded and transcribed verbatim. Interview data were analysed in the same way as data obtained from community forensic staff (see Box 1) using thematic analysis [21].

*Experiential data*. Throughout the research process (original proposed and modified design), reflexive notes were made about general aspects of the research. Brief examples are provided in Table 3, which include issues relating to recruitment challenges and undertaking research in the youth justice system. These notes together with later reflections on observational fieldwork data and personal communications (e.g., emails and telephone calls) were documented. These data were then synthesised alongside the key themes and sub-themes from the thematic analysis of the researcher staff qualitative interview data to examine whether or not these were similar to, or contrasted with, the experiences as documented in interviews.

## Results

The results reported below relate to the modified EBCD methods used to generate young people’s touchpoints from additional sources (i.e., meta-ethnography data) and to identify some of the key challenges of implementing EBCD in youth mental health and/or forensic settings. Table 1 presents four initial touchpoints from young people who offend that were identified from the 14 empirical studies included in the meta-ethnography and through synthesising multiple data sources including observational data, interview, research evidence and theoretical perspectives (see Fig 1). Details of how the four touchpoints might contribute to proposals for service improvement are available elsewhere [17]. Key themes relating to the challenges of implementing EBCD in similar contexts (i.e., youth mental health and/or forensic services) that were identified from interviews with researcher staff, are presented in Table 2. Experiential data from undertaking and reflecting on some of the key challenges experienced in this study are presented in Table 3.

**Table 1:** Young people’s touchpoints.

Touchpoints	Description	Examples
Feeling labelled and living up to expectations	Attitudes expressed by professional staff in forensic services may add strength to the acceptance and permanence (for the young people who offend and staff) of the labels and stigma placed on them.	*‘It’s hard fer me ta change*. *I’m labelled you know… they watch ya more…they’re waiting fer ya ta mess up’* [25]
Feeling uncomfortable about sharing personal information about themselves	Young people’s learned attitudes towards professional staff (e.g., mistrust) together with the risk of potential difficulties in being able to communicate effectively, and a lack of staff training in, and awareness of, particular issues may inhibit young people disclosing information about their particular needs.	*‘Why would I talk about it*? *You’re not supposed to tell people about your personal life’* [26]
Feeling that they are not being listened to	The complex interplay between staff being adequately trained to work with young people who offend, having the appropriate skills and confidence to recognise young people’s vulnerability, and employing coping, may result in missed opportunities to listen to the young people in their care.	*‘I hate them*, *they don’t listen to you… She was saying that I’d be better off away from home and that*. *She didn’t even fucking know me*!*’* [27]
Feeling that they can relate to staff	Whether young people find themselves in the ‘right place at the right time’ and/or where staff are appropriately skilled or ‘matched’ to their needs, may impact on whether or not young people receive support and services that they can relate to.	*‘A few more people telling me ‘no’*. *That’s what would have helped… bit more one-to- one support*. *If I had that I reckon things would have changed*. *Someone I could get on with*, *someone I like*, *someone to look up to really’* [28]

**Table 2:** Researcher staff key themes and sub-themes.

Key themes	Sub-themes	Description	Example quotes
**Developing and maintaining the EBCD collaborative group**	Building relationships	Developing positive working relationships with services through existing links with services, face-to-face meetings, personal contacts and knowledge of the services through having worked previously in these environments, staff who had clearly defined roles within the project (e.g., day-to-day management of the study or participant recruitment) and having a reputable and experienced senior member of staff leading the project.	*‘One thing that helped is that obviously she’s [Principal Investigator] a professional with a lot of experience*. *I think that was something that the service providers like really appreciated and respected’ [ARS3]*
	Creating participation	Research team demonstrating their own commitment to the project, incentivising and tailoring the research to existing service reporting requirements.	*‘I think with every YOT*, *having a focus group*, *if they’re inspected or anything like that*, *it’s always a brownie point’ [SPS3]*
	Maintaining participation	Using different strategies such as regular meetings and updates and developing rapport or drawing on personal relationships with services. Also, knowing when to ‘push’ and when to ‘pull back’ to move the research forward.	*‘So*, *as much as we want to know why the service provider isn’t coming*, *it feels a little uncomfortable sometimes pushing too hard’ [ASR1]*
**Building in flexibility**	Having other options	Exploring other options without compromising the purpose of the research, such as modifying the study protocol (i.e., adjusting the inclusion criteria) or approaching other agencies, and opening up the invitation to young people or staff who had not participated previously or to take part in different stages of the research. Also, drawing on existing sources of data or thinking creatively around how to maximise the use of data already available.	*‘There has to be room and I think there is room in EBCD to modify who the participants are’ [ARS1]*
	Changing circumstances	Flexibility in applying EBCD principles to the study of young people e.g., accepting that over time participants may change their mind and no longer wish to take part, frequent fluctuations within the staff arrangements within service provider organisations, and taking into account that services may change (i.e. young people transitioning between services) and whether to include these.	*‘Sometimes people don’t know it’s their last appointment*. *There’s a whole bunch of…we had a lot of dimensions to consider when we thinking about who is the provider? [ARS3]*
	Timing	Seizing opportunities when young people were formally required to attend services, managing tensions between participant groups around when co-design components could occur, incentivising participation through increasing the honorarium, and negotiating the amount of time set aside for research activities and events.	*‘I just sat in the foyer*. *So when they’d come waiting to see their case worker or what have you*, *I just say*, *“Oh have you got five minutes” and explain what I’m doing’ (ARS2]*
	Capturing data	Recognising the challenges of the different capabilities of young people to engage them in the process and their ability to ‘cope’ with the demands of co-design work, and ‘mixing’ groups e.g., with different diagnoses or attitudes or perspectives. Also acknowledging that the use of film to capture experiences was not always an acceptable method for participants and the need to think about other options e.g., music or art.	*‘We can get the same end results and objectives by doing it different ways…we can do it with music…you will find out all about their life in that rap’ [SPS2]*
**Role of the researcher**	Relationships of power	Recognising that being in a position of holding ‘power’ and making decisions about who was included in EBCD components (e.g., co-design meetings) and where they began (e.g., initial touchpoints that researchers felt were significant to service users) could act as a barrier in the research process. Also, reflecting on the need to include participants (i.e., young people) earlier in the process to draw more on their experiential knowledge and challenge staff’s own assumptions	*‘We often think about what happens as they [young people] get to a service [and] there will be things that they might be able to flag in advance that we won’t’ [ARS1]*
	Emotional work of the researcher	Acknowledging that research studies which involve eliciting the experiences of service users (particularly those who are vulnerable) can be emotionally challenging for both those participating in the research and the researcher and employing strategies to minimise the emotional impact on researchers.	*‘[I] had to try and find a way to keep me and my life and my kids [separate]*, *and not problematise my own circumstances into something that could be’ [ARS2]*

Abbreviations: ARS – Academic Research Staff; SPS – Service Provider Staff.

**Table 3:** Experiential learning from the EBCD methodological study: Some key challenges.

Key Challenge	Documentation
**Maintaining professional relationships with community forensic services**	I received a phone call from a staff member with whom I had been liaising with, to advise me that this was their *‘last day on the job’* due to retirement and they would be leaving the service in *‘the next ten minutes’*. On asking who I should follow-up with, the staff member advised me to contact the senior manager the following week and arrange to meet with their replacement, *‘whoever that was’* [reflexive note]
**Working with different services**	In one YOT, despite the service manager attempting to ‘prioritise’ asking staff to volunteer to participate from their service, the manager felt that competing demands on services and staff resources meant that the interviews were difficult to arrange. During one telephone conversation with a service manager, they sounded noticeably distressed that due to funding and resource issues they had, *‘a caseload pile and no-one to give them to’* [Reflexive note] Arranging custody staff interviews, one senior custody staff member indicated that rather than ask for volunteers, I should *‘tell them I [senior staff member] said they have to’* (reflexive note]
**Accessing young people via gatekeepers**	Opportunities to access young people was challenging. Some staff felt that ‘*It’s too hard – they’re just too hard to engage’ or ‘it’s too time consuming’*. Others stated that *‘they [young people who offend]] wouldn’t want to take part’* [reflexive note]
**Accessing staff**	Seizing opportunities to talk with justice staff often meant working around shift patterns. On one occasion I received an email from a custody sergeant advising me that *‘It is quiet now at [custody suite] if you could get here before 6 [to interview staff]… Next 2 days going to be very busy I suspect at [custody suite]*, *may not be able to spare anyone’* [email correspondence]
**Capturing data**	In discussing the possibility of using film to capture young people’s experiences, one YOT manager described that, *‘their entire current caseload of twelve young men had sexually offended’* and that nature of their offending meant that young people would be reluctant to be filmed or audio recorded due to feelings of shame among their peers and/or lacking trust that their data would kept within the research process and would not be made public, currently or in the future [reflexive note].
**Emotional work involved**	After a particularly emotional interview I emailed the participant to thank them for sharing their valuable knowledge and experiences and to acknowledge that this *‘can be emotional work’*. The staff member responded that it had been a good experience and they were *‘sorry for being a wet blanket’* [email correspondence]. Although the focus of the follow-up contact was the emotional well-being of the participant, I was aware that through observing the participant and listening to their story during the interview I had also been greatly affected by the experience [reflexive note]

## Discussion

This paper describes the problems experienced and the introduction of some EBCD informed innovative methods used in an attempt to facilitate the study of the use of EBCD methodology in community forensic settings with young people who offend and the staff working in these settings. The results provide new knowledge about (i) how these challenges may be linked to particular components of EBCD that require the coming together of participants from different groups (e.g., service providers and service users) across complex and/or multiple services; (ii) some of the practical problems and tensions of applying EBCD to vulnerable populations (e.g. gatekeeping and power imbalances); and (iii) the importance of contextual understanding when evaluating a methodological approach. Understanding the experiences of other research staff involved in similar studies has contributed further evidence about the feasibility of applying and/or modifying the EBCD approach in different settings and with vulnerable population groups.

‘Joint working’ is a key component in the EBCD approach (19). In keeping with previous publications this research highlights that existing on-going problems working with and across agencies (e.g., between UK community youth forensic and young people’s mental health services) was highlighted as a challenge for service providers and also in applying EBCD in these contexts. Some forensic staff were concerned about the time that would be required to commit to the EBCD process and whether or not resources, timetables or shift patterns would allow this. These challenges of working with and across multiple organisations were also reported by research staff who often needed to manage tensions between participant groups about when components of the research would occur as well as incentivising groups to increase their participation. Learning from this methodological study suggests that, recognising and adapting to changes in circumstances (e.g., changing the inclusion criteria and working differently with and/or including additional services), and being supported by a team of researchers that can take responsibility for different components (e.g. building relationships, recruitment and co-design phases) are potentially helpful strategies in being able to complete studies in these community settings.

Although experiences varied, the practical problem of gaining access to vulnerable participants (such as young people who offend) was highlighted in all the different data sources. Legal and ethical requirements placed on particular groups participating in research, e.g., young people in general and certain groups such as young people who offend, mean that designated staff within services often have to take on the role of ‘gatekeeping’ between the young person and the opportunity to take part in a research study [29–31]. In the case of community forensic services, pressures on services to actively and effectively engage with young people and their families in compulsory youth justice work on an involuntary basis, is challenging. In addition to these everyday working responsibilities, attempting and/or being able to voluntarily engage young people in research was often viewed by staff (youth offending team staff) as ‘just too hard’. Further, staff also stated that young people ‘won’t want to take part’. Although working with ‘gatekeepers’ has been described as relatively straight forward in some similar studies reported in this research, ‘gatekeeping’ issues are often considered as a potential barrier to research. This then raises important questions about whether or not staff see a difference between service engagement and research engagement and whether staff involved in focussed roles with young people can break out from their ‘default’ modalities of engagement to do EBCD-type work. Reflecting on this, training for staff might actively promote a more collaborative joint working style as well as building some flexibility in order to effectively support research and service development.

Another finding is the benefit of using alternative data sources in this case the use of observational data, as a means to identify ‘touchpoints’ from young service users when they were unable to directly participate in the research. Nonetheless, Dimopoulos and colleagues argue that although the flexibility of the EBCD can be a strength, adapting the approach can also result in ‘omitting’ key components or stages that are important to co-design [32].

A further key finding from this methodological study and reported previously, relates to understanding knowledge and power dimensions particularly amongst vulnerable participants in specific contexts [33]. A fundamental principle of the EBCD approach is that in order for staff and services users to collaboratively design services collaboratively, relationships of power between these groups may need to be reconfigured [34]. Although professionals working with vulnerable groups must demonstrate care in ‘protecting vulnerabilities’ they must also ‘empower participants’ [33]. Specific to research involving young people who offend, Daykin and colleagues suggest that gatekeepers (i.e., the forensic staff caring for the young people who offend) can not only influence young people’s views but also their access to activities [35]. Furthermore, ‘political ambivalence’ amongst staff in youth forensic services as to whether or not they feel it is appropriate to include the views of young people who offend may also impact on this process [36]. Youth justice has the dual purpose of controlling and punishing youth crime and the responsibility of helping and caring for those young people who offend. These tensions between punishment and welfare can be conflictual for service staff [17, 37]. Yet, decreasing power imbalances between staff and young people is necessary to maximise the likelihood of young people engaging with the youth justice system [38]. Whether or not staff agree with, or are able to, re-negotiate relationships of power therefore raises an important question about whether EBCD is the appropriate methodology to use in these particular contexts and with this group of young people.

Empirical data in this methodological study has highlighted the importance of understanding the context in which EBCD is applied. For this study, the timing of a Government commissioned review created uncertainty about continued employment for Youth Offending Team (YOT) staff, and in some instances to job losses. These higher-level changes occurring in youth justice may have reduced the capacity of services to ‘open the door’ to research and service improvement [33]. Staff needed to prioritise their own workloads over their involvement in and their gatekeeping role to enable young people to participate in the research. Consistent with previous research exploring the processes that can facilitate family carer involvement in EBCD, Chisholm and colleagues report that major organisational change during the study appeared to impact on staff morale and their commitment and ability to prioritise the project [39]. In contrast, researchers undertaking EBCD studies in youth mental health services encountered different organisational fluctuations. Rather than services and staff becoming ‘condensed’, because young people were transitioning between services or moving on to the workplace, additional tasks were need to make, for example, decisions about whether to work with and include additional services, such as organisations supporting young people into employment. Including flexibility to consider alternative options, and/or manage evolving challenges, is probably key to working with young people. These skills are also needed when attempting to enable participation in any kind of research and perhaps especially when embarking on EBCD studies. As discussed previously by Mulvale and colleagues, ‘planning for the unanticipated’ must be considered when integrating and embedding these type of approaches in health (and other) services [33].

### Strengths and limitations

A key strength of this EBCD methodological study is the contribution of new knowledge and evidence about developing the EBCD approach in different contexts with vulnerable population groups. The original proposed study is the first study of its kind to apply the approach in community forensic services and with young people who offend. One limitation of this study is that not all of the components of the research were completed as initially planned. However, this provided an opportunity to further explore how the EBCD approach could be developed and/or modified for this, and similar contexts. In particular, the successful design of a modified approach to include young people’s experiences in participatory service improvement research through identifying touchpoints from secondary data and synthesising other sources of data. This process has generated findings that have the potential to add new insights to the applicability of EBCD to understand the mental health needs of young people who offend. However, further research should consider whether, for example, making significant changes to the traditional EBCD approach and its components may lead to moving too far away from the original character of EBCD [20, 32]. Nonetheless, we believe that this modified approach has the potential to be used in other instances, for example, when researching other sensitive topics or where direct participation may be restricted (e.g., due to legal reasons) and where secondary data may be available. This research has also added new information about the feasibility of this type of research with vulnerable populations through a novel perspective combining three different stakeholders (i.e., young people, staff and researchers). To date the researcher experiences have been relatively unexplored within an EBCD methodology [33]. However, this study has shown that this additional stakeholder perspective can be used to provide new information. Further consideration should be given about how such insights can be woven into a range of modified EBCD approaches.

## Conclusion

This is the first study to utilise an experience-based co-design approach in this context (community forensic services) and with this population group (e.g., young people who offend). In this article, a modified approach to identifying and exploring young people’s experiences (e.g., touchpoints) through secondary analyses of data and observational fieldwork is presented. Data reveal that adopting this type of approach within a context that is perhaps not ‘as ready’ for more ‘traditional’ forms of EBCD, might provide a way to engage with community forensic services and include some information about a group (young people who offend) who were unable to participate directly in the research process.

## Supporting information

S1 File Youth justice staff interview guide. (DOCX)

S2 File Academic and service provider staff interview guide. (DOCX)
