# Supplementary material for: Experience-based co-design (EBCD) with young people who offend: Innovating methodology to reach marginalised groups
Source: PLoS One. 2022 Jul 12;17(7):e0270782. doi: 10.1371/journal.pone.0270782 (PMC9275718; doi:10.1371/journal.pone.0270782)
Supplement: S1 File — (DOCX) [file pone.0270782.s001.docx]

**Supporting Information**

**S1 File. Youth Justice staff interview guide**

# **Staff experiences**

• Can you tell me what is it like working in this service?

• What’s good or not so good about working here?

• What do you think are the main problems with this service from the point of view of staff?

• How does working in this service compare to other places you have worked or are working?

# **Perceptions of young people**

• What do you think it is like being a young person in this service?

• What are your perceptions of the service you are providing to young people?

• Which young people’s needs are met? Not met?

• What do you think are the major problems faced by young people?

• What could be improved for young people in this service?

• In your opinion, what are the major ‘touch points’ or critical moments in a young person’s journey (the things or events that really shaped their overall experience)?

# **Improving the service**

• What do you see are the main priorities for improving the service from the staff point of view?

• What other things do you feel would help to improve your experience and the experience of other staff in this service?

• What do you think young people would identify as things that would help to improve their experiences?

• In your opinion, where might we begin to improve a young person’s experience around this service?
