# Supplementary material for: Experience-based co-design (EBCD) with young people who offend: Innovating methodology to reach marginalised groups
Source: PLoS One. 2022 Jul 12;17(7):e0270782. doi: 10.1371/journal.pone.0270782 (PMC9275718; doi:10.1371/journal.pone.0270782)
Supplement: S2 File — (DOCX) [file pone.0270782.s002.docx]

**Supporting information**

**S2 File. Academic research staff and service provider staff interview guide**

- Can you tell me about your experience of being involved in [TITLE] research project?
- What were some of the challenges you faced when engaging young people in the research?
- What were some of the things that facilitated engagement with young people in the research?
- How do you think we can improve the ways in which young people can be involved in research projects?
